# A Comprehensive Breath Plume Model for Disease Transmission via Expiratory Aerosols

**DOI:** 10.1371/journal.pone.0037088

**Published:** 2012-05-15

**Authors:** Siobhan K. Halloran, Anthony S. Wexler, William D. Ristenpart

**Affiliations:** 1 Department of Chemical Engineering and Materials Science, University of California Davis, Davis, California, United States of America; 2 Department of Mechanical and Aerospace Engineering, University of California Davis, Davis, California, United States of America; 3 Air Quality Research Center, University of California Davis, Davis, California, United States of America; 4 Department of Civil and Environmental Engineering, University of California Davis, Davis, California, United States of America; 5 Department of Land, Air and Water Resources, University of California Davis, Davis, California, United States of America; 6 Department of Food Science and Technology, University of California Davis, Davis, California, United States of America; University of Calgary & ProvLab Alberta, Canada

## Abstract

The peak in influenza incidence during wintertime in temperate regions represents a longstanding, unresolved scientific question. One hypothesis is that the efficacy of airborne transmission via aerosols is increased at lower humidities and temperatures, conditions that prevail in wintertime. Recent work with a guinea pig model by Lowen *et al.* indicated that humidity and temperature do modulate airborne influenza virus transmission, and several investigators have interpreted the observed humidity dependence in terms of airborne virus survivability. This interpretation, however, neglects two key observations: the effect of ambient temperature on the viral growth kinetics within the animals, and the strong influence of the background airflow on transmission. Here we provide a comprehensive theoretical framework for assessing the probability of disease transmission via expiratory aerosols between test animals in laboratory conditions. The spread of aerosols emitted from an infected animal is modeled using dispersion theory for a homogeneous turbulent airflow. The concentration and size distribution of the evaporating droplets in the resulting “Gaussian breath plume” are calculated as functions of position, humidity, and temperature. The overall transmission probability is modeled with a combination of the time-dependent viral concentration in the infected animal and the probability of droplet inhalation by the exposed animal downstream. We demonstrate that the breath plume model is broadly consistent with the results of Lowen *et al.,* without invoking airborne virus survivability. The results also suggest that, at least for guinea pigs, variation in viral kinetics within the infected animals is the dominant factor explaining the increased transmission probability observed at lower temperatures.

## Introduction

Influenza virus transmission rates display a strong peak during wintertime in temperate regions but less defined seasonality in tropical regions, suggesting that environmental factors drive seasonal variations [1], [2]. The exact nature of the underlying environmental factors, however, is unclear. One longstanding hypothesis is that the lower temperatures and lower humidities prevalent in wintertime somehow enhance influenza virus transmission. Recent work by Lowen *et al.* with a guinea pig model [3] provided direct evidence that temperature and humidity do modulate airborne influenza transmission [4]–[6]. They demonstrated that the probability of transmission at low temperatures (5°C) ranged from 100% at low relative humidities to 50% at higher relative humidities. There was more variability at intermediate temperatures, and notably, there was 0% probability of transmission at 30°C regardless of humidity. Lowen *et al.* concluded that both temperature and relative humidity affect influenza virus transmission, but no clear mechanism was identified.

More recently, Shaman and Kohn reexamined the data reported by Lowen *et al.* and demonstrated that the transmission probability was even more strongly correlated with the absolute humidity (i.e., the ambient water vapor pressure) than with the relative humidity [7]. Since the rate of evaporation of airborne droplets depends on the absolute humidity of the air, not the relative humidity, they examined the hypothesis that the probability of transmission was governed by an “aerosol persistence” mechanism. The key idea in this model, which dates back to the pioneering work by Wells and Riley [8], [9], is that larger drops sediment by gravity out of the air more quickly than smaller drops [10], [11]. Accordingly, conditions which favor rapid evaporation (i.e., low absolute humidity) favor the persistence of the droplets in the air and increase the probability of transmission. When comparing Lowen *et al.*'s data against a model predicated on a competition between evaporation and gravitational sedimentation, however, Shaman and Kohn found no correlation [7]. They concluded that aerosol persistence was not responsible for the observed dependence on absolute humidity, and instead they focused on the effect of humidity on virus survivability. Upon reanalyzing a different set of measurements of airborne virus survivability [12], Shaman and Kohn revealed a pronounced correlation between the 1-hour survivability and the absolute humidity. Because of this observation, much subsequent effort has focused on considering the role of airborne virus survivability in explaining epidemiological trends [13]–[17].

There are several pieces of evidence, however, suggesting that virus survivability was not the primary mechanism underlying the transmission behavior observed by Lowen *et al.* First, it is unclear that gravity was the dominant force affecting the motion of the airborne droplets in their experiments. Lowen *et al.* initially focused on establishing that infection occurs between guinea pigs solely by airborne transmission, and they verified that transmission occurs at separation distances between animals up to almost 100 cm away. Crucially, however, they also noted that “…transmission was not observed when the relative positions of the infected and uninfected animals were reversed, suggesting that spread depended on the direction of airflow in the room.” [3] In other words, there was a direct correlation between the direction of airflow and transmission. Subsequent experiments demonstrated the role of airflow even more convincingly, by placing the test animals 100 cm *above* the infected animals, with the airflow directed upward [6]. Disease transmission indeed occurred, obviating any attempt to invoke gravity as the dominant force affecting the motion of pathogen-laden droplets.

A second crucial consideration is that Lowen *et al.* observed a pronounced difference in the viral growth kinetics between animals kept at 5°C and 30°C. Following inoculation, viral concentrations typically increase exponentially during the viral growth period and then decay exponentially as the immune system destroys the virus [18]. The viral concentrations in the guinea pigs tested by Lowen *et al.* followed this pattern, but the animals kept at 5°C exhibited a substantial lag time in the exponential decay. Specifically, the viral concentration in animals kept at 5°C was on average two orders of magnitude larger, for several days, when compared to animals kept at 30°C. Despite the clear difference in the viral growth kinetics within the infected animals, to date no analyses have elucidated the temperature dependence of growth kinetics on the probability of airborne transmission. Given that airborne transmissibility is believed to govern the potential for specific viral strains to spark pandemics [19], [20], a fundamental understanding of both the physics and biology underlying laboratory airborne transmission experiments is imperative.

**Figure 1:**
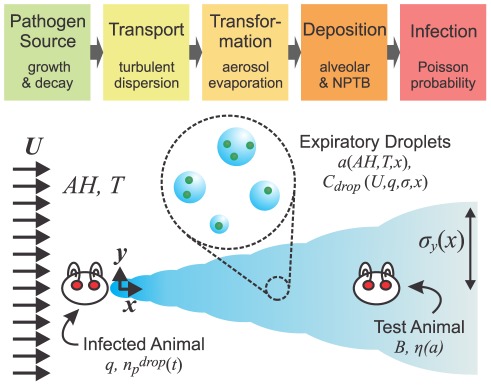
Transmission model and definition sketch. Top, transmission model diagram. Bottom, definition sketch for airborne transmission between animals in a controlled airflow (not to scale). The homogeneous turbulent flow moves left-to-right with average velocity U, turbulent dispersivity (*σ_y_*, *σ_z_*), and fixed humidity and temperature. An infected animal at *x, y* = 0 exhales droplets with pathogen concentration *n_p_^drop^(t)* at a rate *q*. The drops evaporate to size *a_min_*, and disperse via turbulent diffusion to concentration *C_drop_*. The pathogen-laden droplets are potentially inhaled by a test animal downstream that is breathing at rate *B* with deposition efficiency *η(a)*.

In this article we present a comprehensive theoretical framework for assessing the probability of disease transmission via expiratory aerosol droplets between animals placed in a controlled airflow, i.e., a constant background airflow of known direction and velocity. The physics and biology of all five stages of transmission – the pathogen source, transport, transformation during transport, deposition, and infection – are explicitly considered (Figure 1 top). The model yields three important testable hypotheses:

1. The transmission probability will decrease as the airflow velocity increases.
2. The transmission probability will decrease as the degree of turbulence increases.
3. The transmission probability will increase with the *time integral* of the pathogen concentration within the inoculated animal.

We focus here on influenza virus transmission under experimental conditions similar to those used by Lowen *et al.*, although the theoretical framework is applicable to other airborne diseases. A key implication of the model is that temperature-dependent variation in viral growth kinetics within the infected animals, rather than airborne viral survivability, is the dominant factor underlying increased influenza transmission observed experimentally at lower temperatures.

## Methods

### Breath Plume Model

We consider animals placed in individual cages inside an environmental chamber, oriented such that the test animal is placed downstream from the inoculated animal in a horizontal airflow of mean velocity *U* and fixed temperature and humidity (Figure 1 bottom). Although the animals are typically free to move around within their cages, we assume that any fluctuations in position are negligible compared to the distance between animals and that, on average, the test animal occupies a fixed location **x**. The main goal here is to obtain an estimate for the number of pathogens inhaled by the downstream test animal, which necessitates consideration of each of the five stages of transmission (cf. Figure 1 top).

### Pathogen Source

When considering airborne transmission, the first question to ask is: how many pathogens are exhaled by the inoculated animal? This quantity is difficult to measure directly, so instead we obtain insight from consideration of the measured viral concentrations within the animal (typically from nasal titers). Viral infections normally exhibit exponential growth as infected cells produce virus, followed by exponential decay as the immune system deactivates the virus [18]. In the experiments by Lowen *et al.,* the guinea pigs were intranasally inoculated at t = 0, but the test animals were not placed inside the environmental chamber until t = 24 hr; the animals remained within the test chamber for the next seven days.

**Figure 2:**
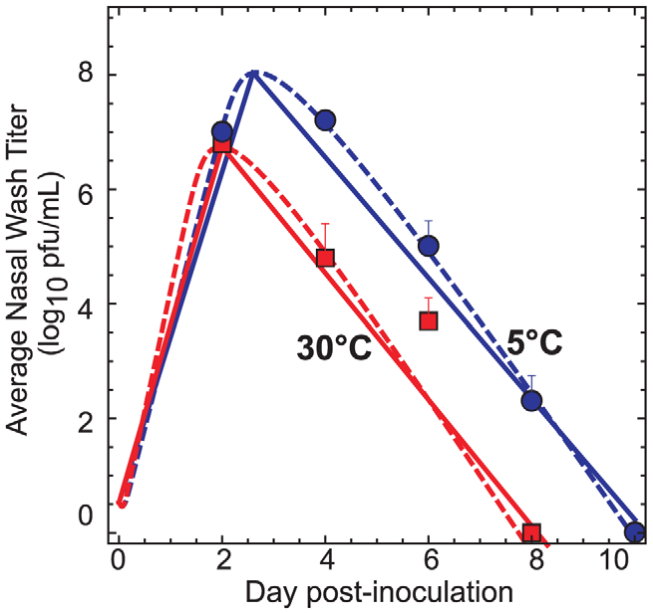
Guinea pig viral growth kinetics at different temperatures. The measurements by Lowen *et al.* [4], [5] of the influenza concentration observed in nasal titers obtained from inoculated guinea pigs maintained at different temperatures. Blue circles: T = 5°C; red squares: T = 30°C. Dashed lines are fits to a numerical model for influenza viral dynamics [18]; solid lines are analytical estimates given by Equation 1.

The viral titers obtained via nasal washes by Lowen *et al.* [4], [5] are plotted in Figure 2 for animals kept at 5°C and 30°C. Qualitatively, the viral concentrations follow the expected behavior, with an initial rapid increase followed by exponential decay. The measured peak viral concentrations were of similar magnitude, but the dynamics are strikingly different: the viral titers of animals kept at 5°C were consistently larger, by about two orders of magnitude, for days 4 through 8. The reason for this difference is unclear; Lowen *et al.* demonstrated that the colder ambient temperature had no measurable effect on the innate immune response of the animals, and they hypothesized that inhalation of colder air somehow favored growth in the mucosa of the guinea pigs [4]. Regardless of the biological mechanism, it is clear that the test animals at the colder ambient temperature were exposed to higher concentrations of virus for a significantly longer time period.

To capture this difference in viral kinetics, we fit Lowen *et al.*'s experimental measurements to a standard model [18] of viral growth (dashed lines, Figure 2). As discussed by Baccam *et al.* [18], experimental measurements of viral concentrations can be fit to a numerical model that tracks the number of healthy or infected target cells (Text S1, Table S2). The viral concentration curves fit using this model make clear that the exponential growth period takes considerably longer and reaches a higher peak concentration at 5°C than at 30°C. A similar observation pertains at 20°C (Figure S1A).

Because both the growth and decay periods are linear on a logarithmic scale [21], a convenient analytical expression may be used to estimate the viral concentrations during the growth and decay, viz.
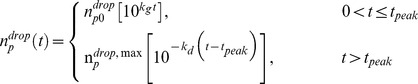
(1)Here, *n_p0_^drop^* is the initial pathogen concentration in the droplet, *n_p_^drop, max^* is the maximum observed concentration in the droplet occurring at time *t_peak_*, and *k_g_* and *k_d_* are the rate constants for the growth and decay periods, respectively. This approach underestimates the numerical solution but allows a purely analytical estimate of the transmission probability (solid lines, Figure 2).

The above discussion focuses on the nasal titers, but the real quantity of interest for airborne transmission is the concentration of pathogens within exhaled droplets. As discussed in detail by Johnson *et al.*, these droplets are most likely formed within the bronchioles via a “film rupture” mechanism [22]. It is well known that viral concentrations within the lungs and the nose can differ substantially; for example, a recent study of influenza viral growth in guinea pig respiratory tracts by Tang *et al.* showed that viral titers varied substantially between the nose, trachea, and lungs, with the nasal titers consistently highest for guinea pigs infected with strains of both H3N2 and H1N1 [23]. Although the absolute concentrations differ, a key observation is that for some strains, the viral titers of the different regions rise and fall in tandem.

Accordingly, we assume that the pathogen concentration within any given expiratory droplet (*n_p_^drop^*) is determined by the pathogen concentration within the bronchial respiratory fluid (*n_p_^bronchial^*) at the time of expiration, which in turn is proportional to the known pathogen concentration within the nasal mucosa (*n_p_^nasal^)*, viz.
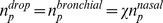
(2)Here *χ* is defined simply as the ratio of the bronchial and nasal pathogen concentrations, which in general could differ for different strains of the same virus. We emphasize that a more direct approach would be to use experimentally measured bronchial pathogen concentrations, but such measurements are non-trivial; in the absence of that information we instead use the above approach based on the nasal titer and a proportionality factor to provide an estimate.

The infected animal is assumed to exhale *q* droplets per unit time, and as a first approximation we assume that *q* is invariant throughout the experiment. The number of pathogens or ‘payload’ in any given droplet is *n_p_^drop^(t)V_O_*, where *V_O_* = 4/3π*a_O_* ^3^ is the initial volume of each droplet upon exhalation. Although the droplet can change size (as discussed below), the pathogens themselves are assumed to be nonvolatile. Accordingly, the number of pathogens in any given drop does not change following exhalation; the time between droplet exhalation by the infected guinea pig and inhalation by the naïve animal downstream is brief, so the pathogens are assumed not to multiply or deactivate during this time period (see *Transformation*). The concentration of pathogens per unit volume of air at any given location is
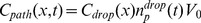
(3)where *C_drop_ (x)* is the local concentration of expiratory droplets per unit volume of air.

### Transport

With an estimate of the pathogen generation in hand, the next question is: how do the pathogen laden droplets move from the inoculated animal to the test animal? Traditionally the concentration *C_drop_* of airborne pathogen-laden droplets has been assumed to be spatially invariant in the surrounding air, i.e., the air in a room is assumed to be well mixed [7]–[9], [11]. Here, however, we are interested in the probability of transmission within airflow occurring in a prescribed direction (cf. Figure 1 bottom). In other words, the air is not well-mixed and the concentration depends sensitively on the location **x** of the test animal with respect to the infected animal. Notably, the guinea pigs used by Lowen *et al.* did not sneeze or cough [3], so here we need not consider the contribution of high-velocity jets due to sneezing and/or coughing on the droplet transport [10], [24]. Instead, we restrict attention to situations where the normal exhalation velocity is small compared to the background airflow; in this limit, the droplets are simply carried along by the background airflow following expiration.

The airflow in typical environmental chambers is turbulent, so upon exhalation the droplets do not simply move in a straight line; the turbulent eddies work to disperse the droplets in directions orthogonal to the mean flow direction. Moreover, if the drops are sufficiently small and the airflow magnitude sufficiently large, then the influence of gravity may be neglected (Text S2). Two archetypal problems have been examined for the case of particulates released from a point source in a uniform turbulent airflow: the ‘puff’ model, in which particulates are released at a discrete point in time, and the ‘plume’ model, in which particulates are released continuously [25]. We are interested here in aerosols released by exhalation in discrete ‘puffs’ from a test animal, so the concentration distribution results from the superposition of multiple puffs. If we restrict attention to time scales much longer than the breathing rate, however, then the periodic variations in the concentration are averaged out and the concentration of droplets within the ‘plume’ is invariant with time. Since the typical breathing frequency is 1 Hz and the typical experiment takes several days, this constraint is readily satisfied.

Accordingly, we invoke the ‘slender Gaussian plume’ model [25] for a point source in a homogenous turbulent flow oriented in the *x*-direction,
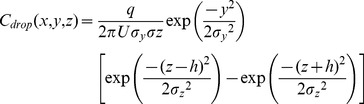
(4)Here *h* is the vertical distance (in the *z*-direction) from the floor to the point source, and the parameters *σ_y_(x)* and *σ_z_(x)* are the root mean square (rms) displacements in the *y* and *z* directions, respectively. In choosing Equation 4, several assumptions have been made. First, we assume that the plume is sufficiently slender such that mean concentration gradients along *x* are negligible compared to those along *y* and *z*, and consequently turbulent dispersion is negligible in the flow direction; this assumption is consistent with the idea that the aerosol displacement is dominated by a large airflow velocity *U*. Second, we assume all droplets impacting the floor are absorbed perfectly such that no droplets ‘bounce’ back into the airflow. Moreover, these impacted droplets are assumed to be non-infective. Finally, we assume that all other solid surfaces (e.g., ceiling, walls) are sufficiently far away that the viral plume is not affected by them; in other words, this approach will be valid provided that the width of the plume is small compared to the dimensions of the chamber.

Similar Gaussian plume models have been considered in the context of airborne disease transmission in outdoor environments, but applications are limited because the model does not account for topographical features, inhomogeneous turbulence, or fluctuations in ambient weather conditions [26], [27]. In contrast, the conditions in a laboratory environment allow greater control over the airflow. Although *σ_y_* and *σ_z_* have not generally been measured for lab-scale environmental chambers with caged animals, experimental work in the context of atmospheric science provides insight on the expected behavior. We assume that the airflow is sufficiently turbulent such that the contribution from molecular diffusivity is negligible, and we restrict attention to short distances sufficiently close to the point source such that the dispersion coefficients grow linearly with distance, viz.,
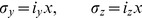
(5)Here *i_y_* = *u_y_/U* and *i_z_* = *u_z_/U*, where *u_y_* and *u_z_* are the rms turbulent velocities in the *y* and *z* directions. These velocities characterize ‘how turbulent’ the flow is and are typically a few percent of the mean velocity [28]. Larger values mean that more material is transported in the orthogonal directions, so concentrations along the center line (*y* = 0, *z* = 0, *x*>0) are reduced.

### Transformation

Next, we ask the question: how do the expired droplets change while they are transported? Following expiration and prior to inhalation by the test animal, the drops and/or the pathogens within them are in general susceptible to changes induced by the ambient conditions. Specifically, the drops may shrink by evaporation, and the pathogens within the drops might become deactivated. Again focusing on the experimental conditions used by Lowen *et al.*, we note that the typical airflow velocity in their environmental chamber is 10 cm/s, which means that only ten seconds transpire before the drops pass a test animal 1 m away. Notably, Harper *et al.* did not make any virus survivability measurements shorter than five minutes [12]; similarly, Hemmes *et al.* reported no survivability measurements shorter than at least two minutes [29]. There is no evidence that virus survivability varies measurably on the time scale of tens of seconds. Accordingly, issues of virus survivability are assumed to not apply on the short time scales of interest here. In other words, the absolute number of pathogens in any given droplet is assumed to remain constant during transport; neither any viral replication nor deactivation occur.

**Figure 3:**
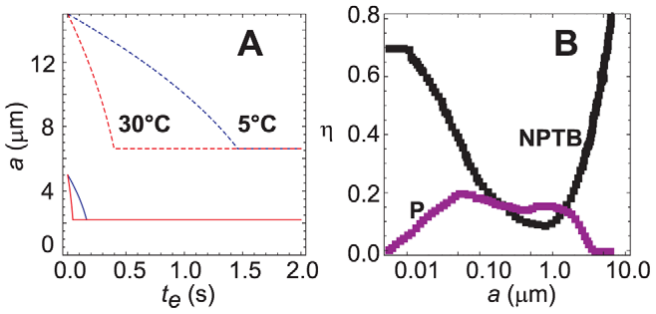
Droplet size evolution and deposition efficiencies. (A) Aerosol size versus time for droplets in air at 50% RH. Solid lines, *a_0_* = 5 µm; dotted lines, *a_0_* = 15 µm. Blue curves: T = 5°C; red curves: T = 30°C. (B) The deposition efficiency of a unit-density particle of radius *a* depositing in the pulmonary (P) and nasopharyngeal-tracheobronchial (NPTB) regions of a guinea pig. Purple: Pulmonary; black: NPTB. Reproduced from Schreider *et al.* [34], [35].

In contrast, the *size* of the droplets is highly sensitive to the ambient temperature and humidity. The droplet size is a concern here not because of gravitational effects (which are neglected), but because droplet inhalation and deposition are highly sensitive to the droplet size (see below). There are many models of droplet evaporation of varying complexity, but here we use the well-known *R^2^* model, so named because the evaporation rate depends on the square of the drop radius. Also used by Shaman and Kohn to estimate the evaporation time [7], the *R^2^* model strictly applies to one-component droplets undergoing pseudo-steady evaporation. However, experimental work by Ranz *et al.* suggests that the *R^2^* model works well even for droplets with large concentrations of proteins or other solutes, until the droplets shrink to a critical size *a_min_* governed by the amount of nonvolatile solute present [30], [31]. This minimum size is given as *a_min_* = *ξa_0_*, where ξ≡*(C_nv_/ρ_nv_)^1/3^* is the ratio of the concentration to density of nonvolatile species in the droplet [32]. The resulting ‘droplet nucleus’ of size *a_min_* will continue to be carried along with the flow. Accordingly, following the standard evaporation analysis [7], droplets shrink with time as
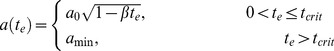
(6)Here *t_e_* is time elapsed since expiration of a droplet with initial radius *a_0_*, and *β^−1^* is a characteristic time scale given by
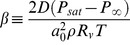
(7)where *D* is the molecular diffusivity of water vapor, *ρ* is the density of liquid water, *R_v_* is the specific gas constant for water, *P_∞_* is the ambient partial pressure of water (i.e., the absolute humidity) and *P_sat_* is the saturation water vapor pressure at the droplet surface. Several features of this model are noteworthy. First, larger values of *β* correspond to shorter evaporation time scales, i.e., the droplet reaches its minimum size more quickly (Figure 3A). As pointed out by Shaman and Kohn, the same relative humidity yields very different evaporation rates at different temperatures. Secondly, the time scale for the droplet to cool down to its final temperature is sufficiently short compared to the time scale for evaporation, so this is regarded as an isothermal process. Also, since we are restricting attention to conditions where the background airflow is invariant in time, and since the droplets are assumed to travel steadily in the x-direction at the same velocity as the airflow, the elapsed time since expiration and droplet position are interchangeable, i.e., *t_e_ = x/U*.

### Deposition

The next key question is: how many droplets are actually inhaled by the test animal? The breathing rates of the infected test animals will vary over the course of a multi-day experiment, due to natural cycles of activity and sleep. As a first approximation, however, we ignore this complexity and assume that *B*, the animal breathing rate, is constant throughout the experiment. Inhalation of the droplet-laden air, however, does not necessarily mean pathogen deposition will occur. The efficiency of deposition is highly sensitive to the droplet size and varies as a function of position throughout the respiratory system [33]. Previous work with airborne transmission has indicated that infections via aerosols are most likely to originate in the lower respiratory tract, specifically the alveoli [32]. In the case of influenza, however, experiments by Lowen *et al.* [3] and Tang *et al.* [23] have shown that infection occurs in both the upper and lower respiratory tract. Both groups investigated regional viral growth kinetics following intranasal inoculation, but to our knowledge, similar experiments have not been conducted in guinea pigs in the context of natural airborne transmission. Because the site of airborne infection is not absolutely clear, we consider here deposition in both the nasopharyngeal-tracheobronchial region (NPTB) and the pulmonary (i.e., alveolar) regions. Typical NPTB and pulmonary deposition efficiency profiles for guinea pigs are plotted versus droplet size in Figure 3B using the model developed by Schreider *et al*. [34], [35].

Note that the deposition efficiency, *η(a)*, involves three key mechanisms: inertial impaction, diffusion, and sedimentation. Figure 3B illustrates that the largest and smallest particles are most likely to deposit in the NPTB region, but by different mechanisms. The increased size of large particles leads to their deposition directly via impaction. In contrast, the motion of small particles is dominated by diffusion; smaller particles diffuse more quickly and are more likely to collide with a surface in the NPTB region and subsequently deposit. Intermediate sized particles are most successful at traversing the airway and depositing in the depths of the lung, i.e., in the alveolar region [34], [35].

The total number of pathogens ultimately deposited in the respiratory system of a test animal during a time interval *dt* is related to the local airborne concentration of pathogens as
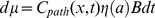
(8)Combination of Equation 8 with Equations 1 and 3 and integration with respect to time yields the expected number of deposited pathogens,
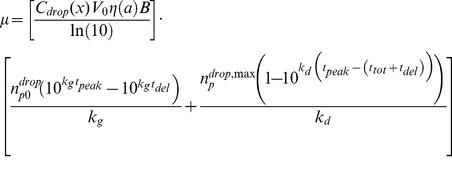
(9)where *t_del_* is the delay time between inoculation and the start of the transmission experiments and *t_tot_* is the total time of exposure. This equation is valid for *t_del_*<*t_peak_*<*t_tot_*+*t_del_*, ensuring the onset of the decay phase occurs during the experiment. As discussed previously, Equation 1 provides an underestimate of the actual pathogen concentration; a more accurate value may be obtained either by integration of the numerical model (cf. dashed lines in Figure 2) or direct numerical quadrature of the experimental measurements. For situations where there is a distribution of initial droplet sizes (as is likely to be encountered experimentally), the total expected value *μ* is simply the sum of the expected values resulting for each size.

### Infection

Finally, we relate the overall probability of transmission to the total number of deposited pathogens in the test animal. We assume that all of the naïve animals in a given experiment are equally susceptible to infection, a condition likely to pertain to genetically similar animals with equivalent health histories. Following the standard approach [32], [36], we assume that the risk of transmission is given by the Poisson probability that the number of deposited pathogens *μ* in the test animal exceeds the number *ν* of pathogens required to initiate infection, i.e.,
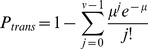
(10)The summation term on the right-hand side represents the probability that fewer than *ν* pathogens deposit. To our knowledge, the minimum infectious dose for guinea pigs is not well characterized, but prior studies have shown that the infectious dose for aerosol transmission in humans is very small [37]. Note that viral titer measurements are typically reported in units of pfu/mL, and it has been previously shown that 1 TCID_50_/mL, and subsequently 1 pfu/mL, likely contains a large number of influenza virions [38], [39]. Accordingly, we assume here that deposition of at least one plaque-forming unit (*ν* = 1) can initiate infection, in which case Equation 10 simplifies to
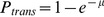
(11)


## Results and Discussion

### Comparison with Experiments

To compare the model with the experimental observations, several parameters require specification. Wherever possible, we used the experimental values reported by Lowen *et al.* To our knowledge, however, the rate *q* and size distribution of expiratory droplets have not been measured for guinea pigs. As an approximation, we instead used measurements from a recent characterization of expiratory droplets released by ferrets intranasally inoculated with influenza [40]. Likewise, the minimum droplet size *a_min_* was estimated using an exhaled breath condensate study conducted by Effros *et al.* [41], who determined the protein and salt concentrations in healthy human respiratory fluid. As for the background airflow, Lowen *et al.* did not report the velocity, but air speed was estimated using known values for flow velocity entering and exiting the Caron Model 6030 environmental chamber. The turbulent dispersivities (cf. Equation 5) were estimated by assuming that the stability class was ‘near neutral’, a condition likely to pertain in controlled laboratory situations [28]. A full list of model parameters is provided in Table S1.

**Figure 4:**
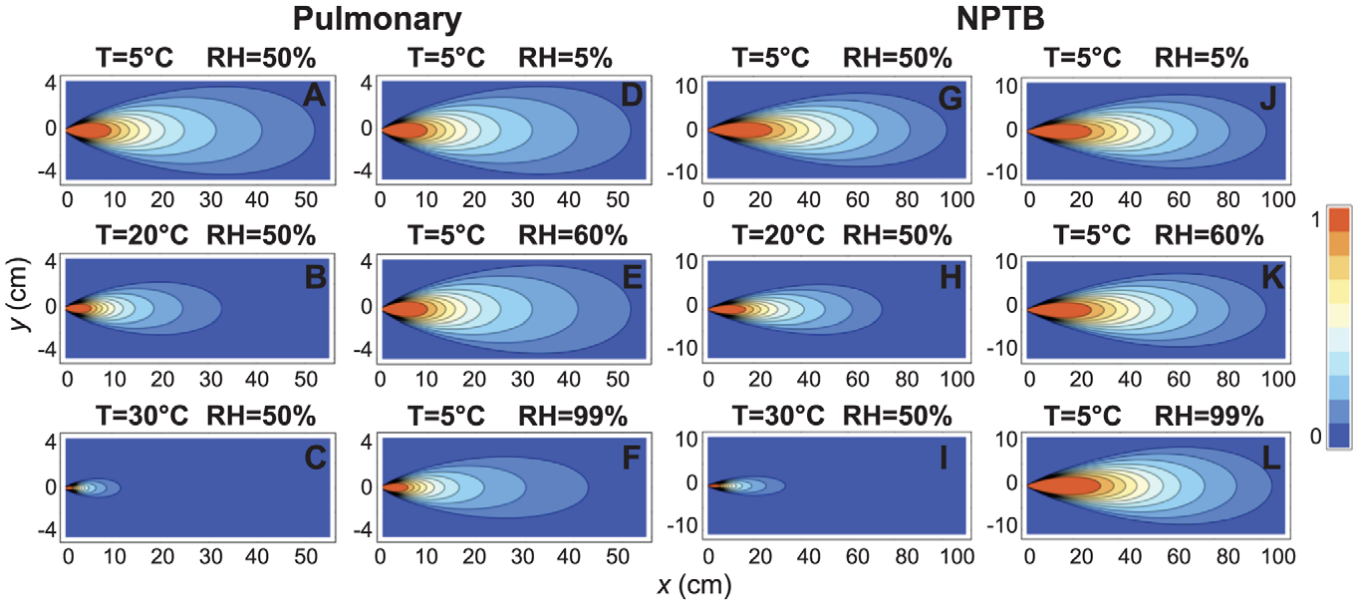
Probability of transmission at different positions. Contour plots of the probability of transmission as a function of position downstream from an infected animal located at the origin for the pulmonary (A–F) and NPTB (G–L) deposition efficiencies. Red denotes high probability of transmission, blue denotes low probability. (A–C, G–I) Fixed relative humidity and varying temperature. (D–F, J–L) Fixed temperature and varying relative humidity. Note that the transmission probability depends strongly on temperature but more weakly on humidity.

Representative contour plots of the transmission probability as a function of downstream position, as calculated by Equation 11, are presented in Figure 4 for different temperatures and humidities for both the pulmonary and NPTB deposition efficiencies. In each case there is a high probability of transmission near the inoculated animal (i.e., near *x, y* = 0, 0), with the probability decaying toward zero at larger distances. The exact shape of the ‘infectious zone’, however, depends sensitively on the ambient temperature for both deposition efficiencies (Figure 4A, B, C, G, H, I): at 5°C the infectious zone extends nearly five times as far as at 30°C. The effect of humidity is more subtle. There is no significant difference between 5% and 60% relative humidity at 5°C (Figure 4D, E, J, K), but at sufficiently high humidity, the size of the infectious zone decreases appreciably for the pulmonary deposition model (Figure 4F). At high humidity, the infectious zone is essentially the same size as at lower humidities for the NPTB deposition efficiency, but there is a slight increase in probability of infection very close to the inoculated guinea pig (Figure 4L). We emphasize that the temperature here affects only the viral growth kinetics within the infected animals (cf. Figure 2) and the rate of droplet evaporation (cf. Figure 3A), while the humidity only affects the rate of evaporation. The probability contours presented in Figure 4 can thus be interpreted in terms of how the temperature and humidity affect the ‘payload’ of pathogens delivered to the test animal. The range of infection is increased at low temperatures primarily due to the longer time period of increased viral concentrations emitted by the animal; the growth period is extended and the peak viral titer is higher, so more pathogen is released. At the higher temperatures, in contrast, the viral concentrations in the animal decay more rapidly and have a lower peak value so less pathogen is transmitted, decreasing the probability of transmission.

The pathogen payload also depends sensitively on the droplet size upon inhalation by the test animal. A key point is that larger droplets carry a much larger number of pathogens (a higher payload), but are also more likely to deposit in the NPTB region than the alveoli (cf. Figure 3B). The primary role of evaporation, then, is to shrink the larger droplets to a size that has a higher probability of traversing deep into the airway and depositing within the lower respiratory tract of the test animal. Under many conditions, however, the evaporation proceeds so rapidly that all drops reach their terminal size within a very short distance from the source. This effect can be seen in Figure 4D, E and J, K, where the transmission probability contours barely change despite a 37% reduction in the evaporation rate. Only at very low evaporation rates, such as at 99% RH (Figure 4F, L), do the droplets fail to reach a size appropriate for deposition in the alveoli within a sufficiently short distance. Although the droplets eventually reach their terminal size even at high humidities, they are so far downstream that the turbulent dispersion has diluted the effective droplet concentration to negligible values.

**Figure 5:**
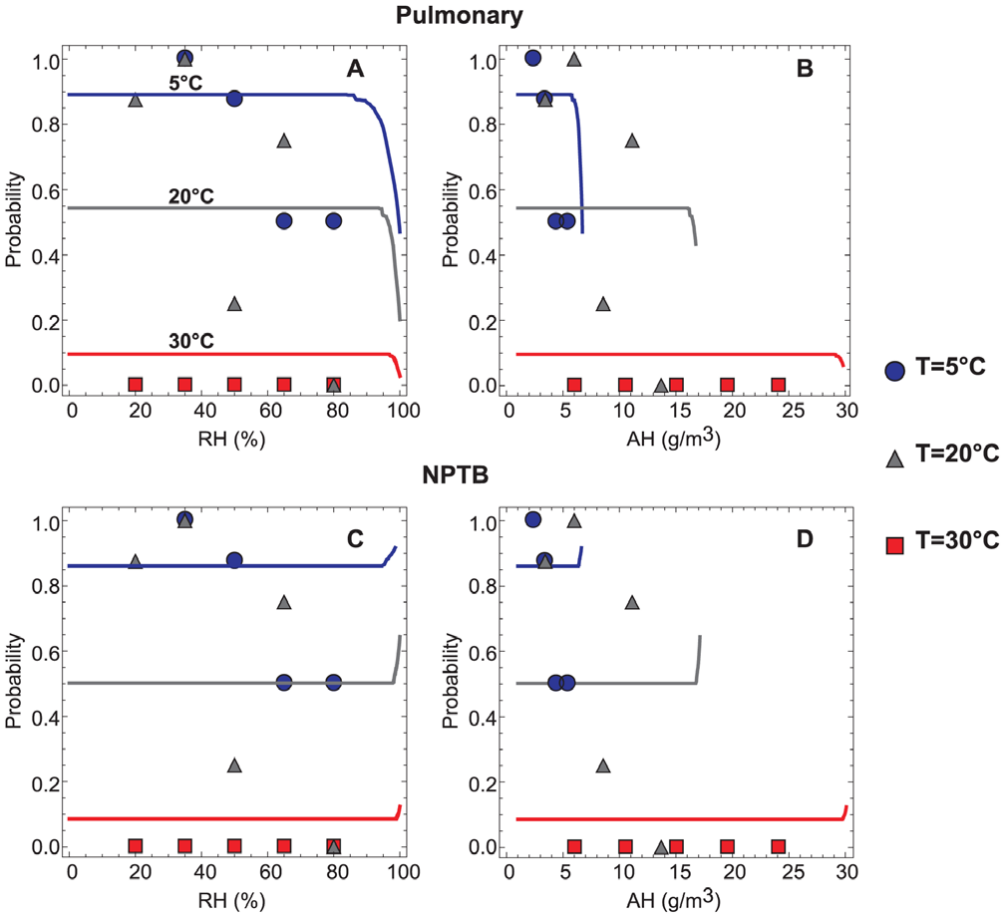
Effect of ambient humidity and temperature on transmission probability. The predicted probability of transmission at varied temperatures versus (A, C) relative humidity and (B, D) absolute humidity for the pulmonary (A–B) and NPTB (C–D) deposition efficiencies at 10 cm and 30 cm downstream, respectively. The experimental observations by Lowen *et al.* [4], [5] are shown as discrete points. Blue circles: T = 5°C; gray triangles: T = 20°C; red squares: T = 30°C.

The model is compared directly to the experimental measurements by Lowen *et al.* in Figure 5. Note that one of the main implications of the model is that simple linear regressions of transmission probability versus either temperature or humidity will not yield strong correlations, since the temperature serves as a confounding variable for humidity. Instead, both temperature and humidity should be considered simultaneously. Figure 5 presents the predicted probability of transmission at a fixed distance downstream as a function of relative humidity and absolute humidity for varied temperatures for the alveolar and NPTB deposition models. The experimental measurements by Lowen *et al.* are superimposed as discrete points. The model qualitatively captures two important trends. First, the predicted probability of transmission is close to zero at 30°C regardless of humidity, in accord with the experimental observations, for both deposition models. Second, the model predicts the highest probability of transmission at 5°C and lower humidities for the alveolar model (Figure 5A–B). At very high relative humidities, probability drops off rapidly at all temperatures as the evaporative process is slowed. For the NPTB model, there is a slight increase in probability at very high relative humidities, as the likelihood of deposition here increases with size.

**Figure 6:**
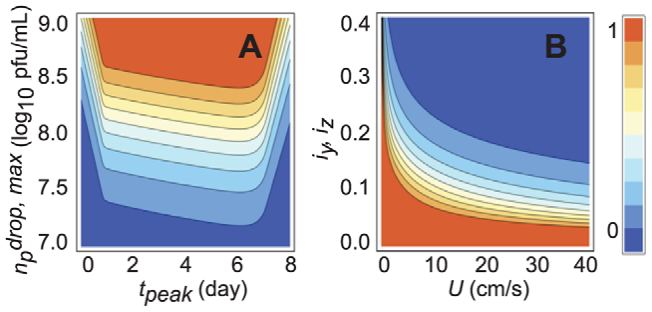
Sensitivity analysis of viral kinetics and airflow parameters for NPTB deposition efficiency 30 cm downstream. (A) Contour plot of transmission probability as a function of *n_p_^drop, max^* and *t_peak_*. The animals are assumed to be brought into contact one day post-inoculation and removed seven days later. (B) Contour plot of transmission probability as a function of the turbulent dispersivity coefficients *i_y_*, *i_z_* (assumed equal) and mean airflow velocity *U*. Small changes in either the degree of turbulence or the flow velocity yield large changes in the transmission probability.

One of the key implications of the model is that the probability of infection depends very sensitively on both the viral growth dynamics within the inoculated animals and the physical details of the airflow. This former effect is explored in Figure 6A, which shows the probability of transmission for varied *n_p_^drop, max^* and *t_peak_* (all other variables held constant). As noted in the introduction, the transmission probability depends directly on the integral with respect to time of the viral concentration within the inoculated animal; the time integral itself depends on *n_p_^drop, max^* and *t_peak_*. Two trends are apparent in Figure 6A. First, the likelihood of infection is increased for higher values of *n_p_^drop, max^*, as expected intuitively. Figure 6A makes clear, however, that a factor of 10 reduction in the peak pathogen concentration can significantly reduce the probability of transmission; note that the probability drops from nearly 100% to about 10% for a reduction from log*n_p_^drop, max^* = 8.5 to 7.5. The effect of varied *t_peak_* is more subtle. At low values of *t_peak_*, the onset of the decay period occurs prior to exposure of the naïve animal, and the peak concentrations are missed. At high values of *t_peak_*, the onset of the decay period occurs after the experiment is finished, but *k_g_* is lower, again decreasing the total amount of pathogen to which the naive guinea pig is exposed. For intermediate values of *t_peak_*, the probability increases weakly with increased *t_peak_*.

The effect of the airflow parameters on the transmission probability (for fixed viral growth parameters) is shown in Figure 6B. For animals located along *y* = 0, increasing the turbulent dispersivity slightly causes significant reductions in the transmission probability. Physically, this decrease reflects the increased dispersion of droplets in the orthogonal direction; in other words, increasing the turbulence spreads the droplets further away from the test animal, decreasing the number ultimately inhaled. Similarly, increasing the mean airflow velocity decreases the transmission probability because the droplets blow by so quickly that the concentration at any point is lowered (Equation 4). We note that limited data exists to corroborate the effect of airspeed predicted here; specifically, the transmission probability was observed to decrease with increased airflow velocity in an early study with mice [42]. This result and Figure 6B demonstrate that accurate measurements of the airflow velocity and degree of turbulence are crucial in airborne transmission experiments, since small changes in the nature of the airflow might account for differences in transmission probability that would otherwise be erroneously attributed to differences in biological infectiousness.

**Figure 7:**
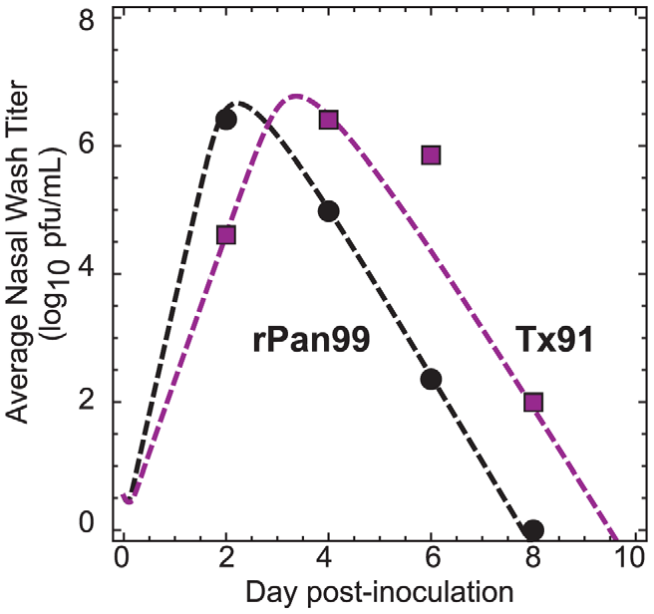
Guinea pig viral growth kinetics of rPan99 and Tx91. The measurements by Mubareka *et al.* [6] of the influenza concentration observed in nasal titers obtained from inoculated guinea pigs infected with rPan99 and Tx91. Black circles: rPan99; purple squares: Tx91. Dashed lines are fits to a numerical model for influenza viral dynamics [18].

**Figure 8:**
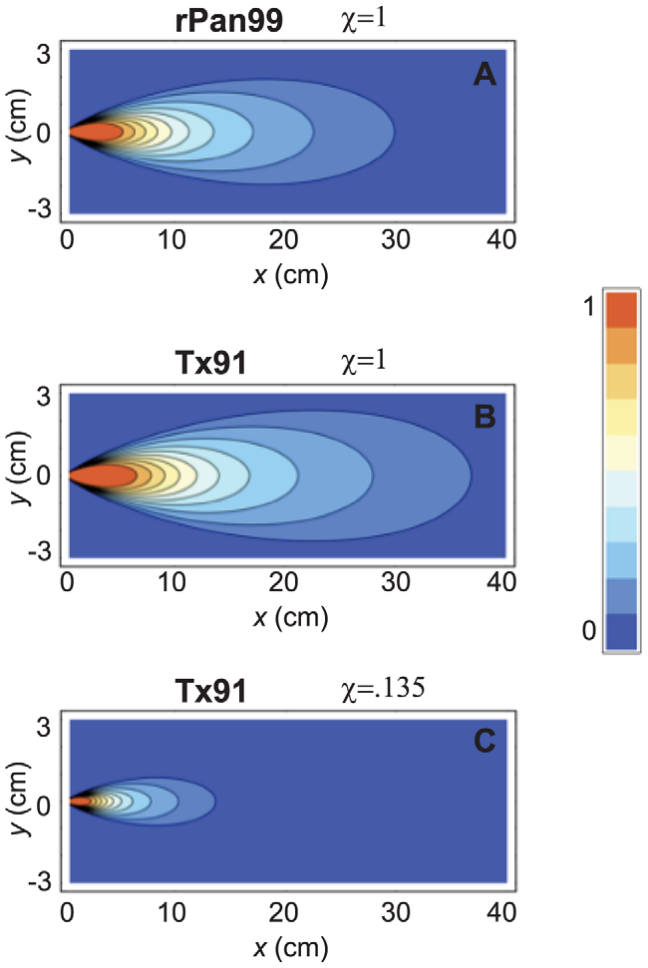
Probability of transmission at different positions for rPan99 and Tx91 experiments. Contour plot of transmission probability in (A) rPan99 experiment with χ = 1, (B) Tx91 experiment with χ = 1, and (C) Tx91 experiment with χ = .135 using the NPTB deposition efficiency. At x≈7 cm, transmission probabilities match the findings of Mubareka *et al.* [6] (A, C).

Finally, we consider the effect of χ, the ratio of the nasal titer to bronchial titer (cf. Equation 2). Lowen *et al.* predominantly studied a strain of H3N2, influenza A/Panama/2007/99 (Pan99) [3]–[5]. In a more recent paper, Mubareka *et al.* also looked at airborne transmission with a strain of H1N1, influenza A/Texas/36/1991 (Tx91). The viral kinetics of guinea pigs intranasally inoculated with Tx91 and rPan99, a recombinant form of Pan99, are shown in Figure 7 [6]. The viral kinetics within guinea pigs inoculated with each strain follow the standard exponential growth and decay patterns, but the growth phase in the Tx91 inoculated guinea pig takes slightly longer. Figure 8 shows that the infectious zone for the Tx91 infected guinea pig (Figure 8B) is slightly larger than that of the rPan99 strain (Figure 8A) when χ for both strains is assumed to be 1. However, Mubareka *et al.* reported higher transmissibility for rPan99 than for Tx91 in airborne experiments; 75% (3/4) of guinea pigs became infected with rPan99, while only 25% (1/4) contracted Tx91 [6]. Mubareka *et al.* measured the airborne titers of each strain using an aerosol sampling technique, and found that the maximum concentration of rPan99 was nearly an order of magnitude greater than the largest measurement of Tx91 [6].

We emphasize that the above observation supports the central assumption of the model presented here: the higher the airborne concentration of pathogens, the higher the probability of transmission. It also highlights, however, the necessity of measuring the pathogen concentration within the respiratory fluid at the most likely point of origin of the expiratory aerosols, i.e., the bronchioles. Specifically, Mubareka *et al.*'s findings [6] suggest that the bronchial concentration of Tx91 virus and the corresponding concentration in the exhaled droplets are lower compared to rPan99. Since the nasal titers for guinea pigs infected with the two strains were roughly equivalent, the observations suggest that the proportionality constant χ is smaller for Tx91 infected guinea pigs than for rPan99 infected animals. Indeed, our model calculations suggest that a small change in χ yields a significant change in transmission probability: a decrease in χ from 1 to 0.135 yields a decrease in transmission probability from 88% to 25% (Figure 8B–C). Note that a factor of 6 difference between nasal and bronchial concentration is quite possible, given for example the observations for virus growth in the nose and lungs reported by Tang *et al*. [23] Since χ has not yet been measured experimentally, it serves here as a fitting parameter; the key result however is that plausible values of χ yield results that are consistent with the observed effect of different viral strains. In the context of the experiments of Mubareka *et al.,* varying χ illustrates the difference in airborne transmissibility of different strains of influenza A. The model can be also used to show that different strains can have the same χ value and thus have similar transmission patterns, as was the case in the recent experiments of Steel *et al.* [43] (Text S4, Figures S2 and S3).

### Model Implications

The theoretical framework developed here suggests a number of future experiments. First, assessments of transmission probability will greatly benefit from higher measurement frequency during the growth stage of the viral dynamics. The earliest measurements by Lowen *et al.* of the viral concentration did not occur until 48 hours post-inoculation, so the amount of pathogen the animals are exposed to during the first 24 hours must be inferred by numerical modeling. Experimental measurements at earlier times near *t_peak_* are crucial, as well as careful determination of the droplet expulsion rate and droplet composition, including the number of pathogens per droplet. Other key infective parameters, such as the minimum infectious dose, should be assessed. Moreover, exhaled breath condensate studies from guinea pigs infected with each virus will be essential to gauge the actual number of exhaled pathogens as a function of time following inoculation. Second, both the experiments and the model clearly indicate that the airflow greatly affects the transmission probability, but to date no experimental characterization of the background airflow has been performed. The cages and the guinea pigs themselves could also alter the airflow, as they provide physical barriers within the chamber; flow profiles around physical obstructions should be experimentally measured to gauge their impact on transmission.

Third, variability in the guinea pig breathing rate should be characterized. Minute ventilation, the amount breathed by the animal per minute, has a strong temperature dependence [44], but to our knowledge, has not been investigated for guinea pigs. This relationship is important for determining both the number of respiratory droplets released into the air (*q*) and the amount of air inhaled by the test animal (*B*). The effect of ambient humidity should likewise be measured, since humidity also affects the breathing rate [44]. Similarly, the activity level of the guinea pig will cause changes in its breathing patterns; respiration experiments should be conducted in a number of scenarios, with animals at rest and in motion.

Fourth, the evaporation model used here should be tested rigorously with mucosa from infected animals. The nonvolatile materials in the expelled respiratory aerosols will depress the vapor pressure of the water, thus slowing the evaporation process and altering the probability distribution. The aerosols may also form a permanent solid structure prior to releasing all of their water [30], [31]. At higher relative humidities, the hygroscopic nature of the salt components may cause the droplets to increase in size. At relative humidities greater than the deliquescence relative humidity, aerosols will begin to grow through the acquisition of water from their surroundings [32]. Conversely, at the efflorescence relative humidity, aerosols will suddenly expel their water content and crystallize. The value of the efflorescence and deliquescence relative humidities are not known due to the droplets' complex and varying chemical compositions, so the range of relative humidities during which evaporation will occur versus growth or crystallization has not been identified.

Fifth, the effect of humidity on the viral growth kinetics within the animal should be investigated. As shown in Figure 4, the model predicts an increase in NPTB infection at high humidity, whereas experimentally a decrease in transmission is observed. An important caveat is that the model presented here only considers the effect of ambient humidity on the evaporation rate of the droplets. If humidity affects anything else – such as the viral growth kinetics – then the model must be extended to include those effects. Additional experimental work should be performed at constant temperature but varied humidity to directly assess whether humidity modulates the viral growth kinetics or some other key parameter in the model (e.g., deposition efficiency or breathing rate).

Although we focus here on influenza A, the theoretical framework is applicable to any sort of airborne disease carried by expiratory droplets (e.g., pneumonia, measles, smallpox, or the common cold). The model could be expanded to account for increased droplet concentration created through symptoms such as sneezing and coughing, which typically generate a greater number of droplets with a significantly larger average size compared to those generated by regular breathing [45, Text S3]. Different deposition models could be employed, especially when looking at infection in different species. Likewise, since the animals are typically mobile in their cages, information about the dynamics of their relative distances from each other could be included. Furthermore, the model presented here assumes air is not recirculated through the test chamber. Since national standards for animal care have a high fresh air requirement, it is likely the recirculated air is highly diluted. If a substantial amount of air is recirculated, however, then details about the frequency with which old air is removed and fresh air is introduced must be included, as well as the filtration coefficient, which quantifies the percent of droplets removed from the air per pass through the chamber (i.e., by contact with any dust filters or airflow equipment). If a sufficiently large amount of air is being recirculated with minimal filtration, virus survivability could also become an important factor.

As a final comment, the basic framework of the model could be extended to case of airborne transmission between humans, but several challenges must be overcome. First, the droplet expiration associated with talking, coughing, and sneezing must be incorporated [46], [47]; these processes all generate a large number of expiratory droplets, which contain infective pathogens [48]–[52]. A more general model would also need to be adjusted to account for differences in immunological response, including pre-existing immunities. Most importantly, humans tend to be much more mobile than animals in cages, so even in situations where humans are located in a time-invariant background airflow (e.g., the air-conditioning in an office or classroom environment), information about the dynamics of the relative distance between infected and uninfected individuals would need to be explicitly included. The theory presented here serves as a framework for considering these more complicated effects.

## Supporting Information

Figure S1 **Viral kinetics and expelled particle size distribution.** (A) The measurements by Lowen *et al.* [4], [5] of the influenza concentration observed in nasal titers obtained from inoculated guinea pigs maintained at different temperatures. Blue circles: T = 5°C; gray triangles: T = 20°C. Dashed lines are fits to a numerical model for influenza viral dynamics [18]; solid lines are analytical estimates given by Equation 1. (B) Size distribution of respiratory particles from normally paced closed mouth respiration from ferrets infected with influenza A/Panama/2007/99 (Pan99). Reproduced from Gustin *et al.* [40].
(TIF)

Figure S2 **Guinea pig viral growth kinetics of Pan99 and Tx91.** The measurements by Steel *et al.* [43] of the influenza concentration observed in nasal titers obtained from inoculated guinea pigs infected with Pan99 and NL09 housed at (A) 20°C and (B) 30°C. Black circles: Pan99; green squares: NL09. Dashed lines are fits to a numerical model for influenza viral dynamics [18].
(TIF)

Figure S3 **Probability of transmission at different positions for Pan99 and NL09 experiments.** Contour plot of transmission probability for ambient conditions used in the experiments of Steel *et al.* [43] (A–B) T = 20°C, RH = 65%. (C–D) T = 20°C, RH = 80%. (E–F) T = 30°C, RH = 20%. (G–H) T = 30°C, RH = 80%. Temperature and humidity trends for both deposition models were consistent, so results are shown simply for the NPTB deposition model. For both strains, χ = 1. Infection rates reported by Steel *et al.* [43] are listed above each plot.
(TIF)

Table S1 **Model Parameters.**
(PDF)

Table S2 **Viral Kinetics Model Parameters.**
(PDF)

Text S1 **Viral Kinetics Model.**
(DOC)

Text S2 **Justification for Neglecting Gravity.**
(DOC)

Text S3 **Respiratory Particle Size Distribution.**
(DOC)

Text S4 **Comparison with Steel** ***et al.*** **experiments.**
(DOC)
