# Supplementary figures and images for: A Comprehensive Breath Plume Model for Disease Transmission via Expiratory Aerosols
Source: PLoS One. 2012 May 15;7(5):e37088. doi: 10.1371/journal.pone.0037088 (PMC3352828; doi:10.1371/journal.pone.0037088)

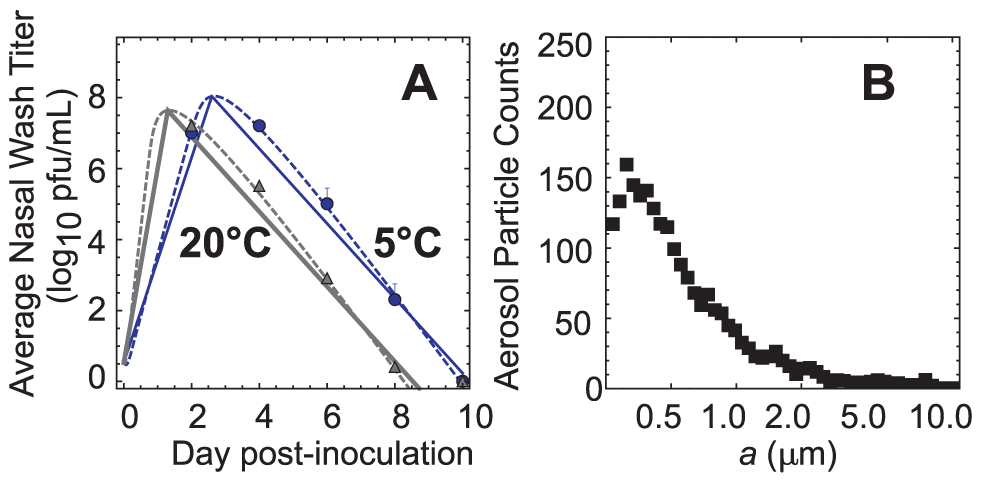

Supplement: Figure S1 — Viral kinetics and expelled particle size distribution. (A) The measurements by Lowen et al. [4], [5] of the influenza concentration observed in nasal titers obtained from inoculated guinea pigs maintained at different temperatures. Blue circles: T = 5°C; gray triangles: T = 20°C. Dashed lines are fits to a numerical model for influenza viral dynamics [18]; solid lines are analytical estimates given by Equation 1. (B) Size distribution of respiratory particles from normally paced closed mouth respiration from ferrets infected with influenza A/Panama/2007/99 (Pan99). Reproduced from Gustin et al. [40]. (TIF) [file pone.0037088.s001.tif]

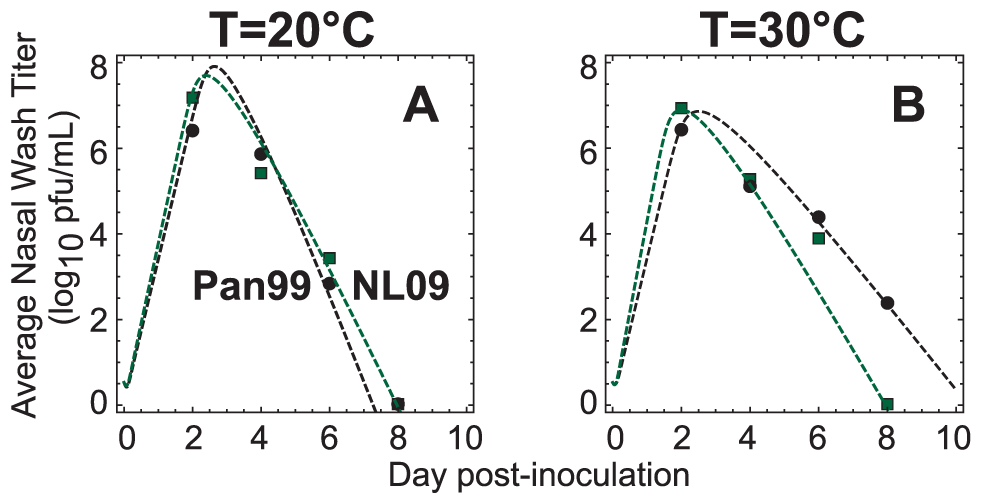

Supplement: Figure S2 — Guinea pig viral growth kinetics of Pan99 and Tx91. The measurements by Steel et al. [43] of the influenza concentration observed in nasal titers obtained from inoculated guinea pigs infected with Pan99 and NL09 housed at (A) 20°C and (B) 30°C. Black circles: Pan99; green squares: NL09. Dashed lines are fits to a numerical model for influenza viral dynamics [18]. (TIF) [file pone.0037088.s002.tif]

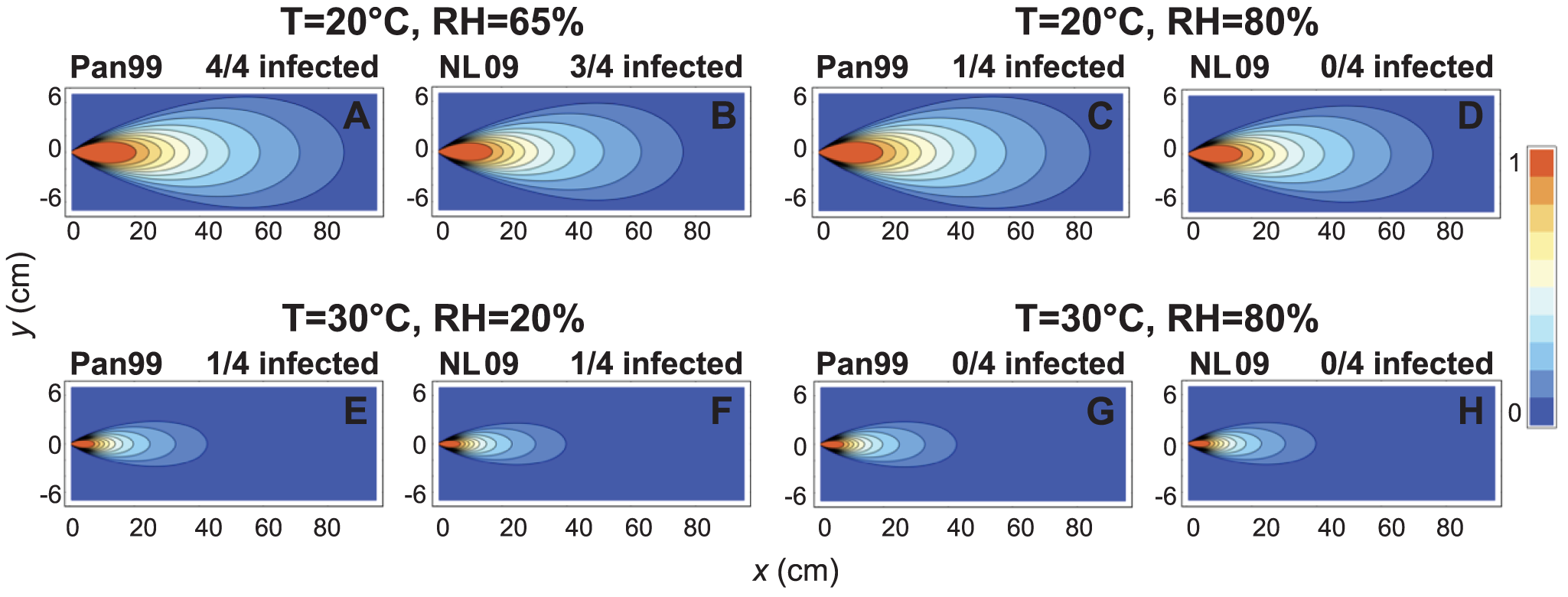

Supplement: Figure S3 — Probability of transmission at different positions for Pan99 and NL09 experiments. Contour plot of transmission probability for ambient conditions used in the experiments of Steel et al. [43] (A–B) T = 20°C, RH = 65%. (C–D) T = 20°C, RH = 80%. (E–F) T = 30°C, RH = 20%. (G–H) T = 30°C, RH = 80%. Temperature and humidity trends for both deposition models were consistent, so results are shown simply for the NPTB deposition model. For both strains, χ = 1. Infection rates reported by Steel et al. [43] are listed above each plot. (TIF) [file pone.0037088.s003.tif]
