# Supplementary material for: A Comprehensive Breath Plume Model for Disease Transmission via Expiratory Aerosols
Source: PLoS One. 2012 May 15;7(5):e37088. doi: 10.1371/journal.pone.0037088 (PMC3352828; doi:10.1371/journal.pone.0037088)
Supplement: Table S1 — Model Parameters. (PDF) [file pone.0037088.s004.pdf]

Table S1: Model Parameters

	Parameter	Value	Reference
General	TV_{gp}	4.4 cm^3/breath	[1]
	TV_{human}	500 cm^3/breath	[2]
	BR_{gp}	60 breaths/min	[1]
	BR_{human}	12 breaths/min	[2]
	h	4 cm	[3]
	ρ	.999 g/cm^3	[4]
Airflow	U	10 cm/s	[5, 6]
	u_y	.75 cm/s	[7]
	u_z	.75 cm/s	[7]
	T	278-303 K	[5, 6]
	RH	0-100 %	[5, 6]
Infectivity	k_g	2-5 d^{-1}	[5, 6]
	k_d	1-2 d^{-1}	[5, 6]
	t_{tot}	7 d	[5, 6]
Aerosol	q	1.19 s^{-1}	[1, 8]
	a_0	.27-8.04 μm	[8]
	ξ	0.44	[9]
Respiratory	B	3.83 x 10^5 cm^3/d	[1]
	$\eta(a)$	see Deposition section	[1]

References

1. Schreider JP, Hutchens JO (1979) Particle deposition in the guinea pig respiratory tract. J Aerosol Sci 10:599–607.

2. Sherwood L (2005) Fundamentals of Physiology: A Human Perspective. New York: Brooks Cole. 736 p.

3. Terril L, Clemons D (2005) The Laboratory Guinea Pig. New York: CRC-Press. 192 p.

4. Mills AF (1995) Fundamentals of Physiology: A Human Perspective. New York: CRC-Press. 1240 p.

5. Lowen AC, Mubareka S, Steel J, Palese P (2007) Influenza virus transmission is dependent on relative humidity and temperature. PLoS Pathog 3:1470–1476.

6. Lowen AC, Steel J, Mubareka S, Palese P (2008) High temperature (30°C) blocks aerosol but not contact transmission of influenza virus. J Virol 82:5650–5652.

7. Csanady GT (1973) Turbulent Diffusion in the Environment. Boston: D. Reidel Publishing Company. 72 p.

8. Gustin KM, Belser JA, Wadford DA, Pearce MB, Katz JM, et al. (2011) Influenza virus aerosol exposure and analytical system for ferrets. Proc Natl Acad Sci USA 106:8432–8437.

9. Nicas M, Nazaroff WW, Hubbard A (2005) Toward understanding the risk of secondary airborne infection: Emission of respirable pathogens. J Occup Environ Hyg 2:143–154.
