# Supplementary material for: A Comprehensive Breath Plume Model for Disease Transmission via Expiratory Aerosols
Source: PLoS One. 2012 May 15;7(5):e37088. doi: 10.1371/journal.pone.0037088 (PMC3352828; doi:10.1371/journal.pone.0037088)
Supplement: Table S2 — Viral Kinetics Model Parameters. (PDF) [file pone.0037088.s005.pdf]

Table S2: Viral Kinetics Model Parameters

T, °C	β, [(pfu/mL) d]$^{-1}$	k, d^{-1}	p, (pfu/ml)/d	δ, d^{-1}	c, d^{-1}	V_i, pfu/mL	T_i, #
5	3.04×10^{-7}	4	20.7	3.1	3.1	3.38	6.05×10^{7}
20	6.52×10^{-6}	3.5	8.54	3.5	3.0	3.38	6.05×10^{7}
30	7.97×10^{-6}	4	1.24	3.1	4.1	3.38	6.05×10^{7}
