# Supplementary material for: A Comprehensive Breath Plume Model for Disease Transmission via Expiratory Aerosols
Source: PLoS One. 2012 May 15;7(5):e37088. doi: 10.1371/journal.pone.0037088 (PMC3352828; doi:10.1371/journal.pone.0037088)
Supplement: Text S1 — Viral Kinetics Model. (DOC) [file pone.0037088.s006.doc]

**Supporting Information**

**Text S1**

**Viral Kinetics Model**

To quantify the concentration of virus in the respiratory fluid of the infected guinea pigs throughout the exposure period, we used a target cell-limited model that accounts for a lag time between infection and virus production [1]. The target cell-limited model is one of the simplest methods of describing viral kinetics because it does not contain a complex immune response; the kinetics are instead dictated primarily by the number of susceptible target cells. This situation pertains when the primary adaptive immune response occurs substantially after the peak viral concentration is observed, i.e., when most of the virus is already depleted [2]. This target-cell limited model has been previously used to describe viral kinetics in human subjects infected with H1N1 influenza A virus [1, 3]. In both these experiments and those of Lowen *et al*., subjects were infected intranasally, likely leading to viral growth in the upper respiratory tract [1].

The target cell-limited model is expressed by the following set of coupled ordinary differential equations:

$$\frac{dT}{dt}=-\beta TV$$

$\frac{dI_{2}}{dt}=kI_{1}-\delta I_{2}$

$\frac{dV}{dt}=pI_{2}-cV$ **(S1)**

Here *T(t)* is the number of healthy target cells, *β* is the rate constant for infection of these cells, *V(t)* is the viral titer, *I_1_(t)* is the number of infected target cells that are not yet producing virus, *k* is the rate constant for infected target cells to begin producing virus, *I_2_(t)* is the number of infected target cells that have begun producing virus, *p* is the rate at which infected cells produce new virus particles, and *δ* and *c* are the rates of cell death and virus clearance, respectively. The immune response is implicitly incorporated through *δ* and *c*. *T_i_*, the number of target epithelial cells available for infection in the nasal turbinates, was estimated by multiplying *T_i_* for humans [1] by a scaling factor accounting for the difference in the surface area of the human nasal tract and the guinea pig nasal tract [4]. The parameters *β*, *k*, *p*, *δ,* and *c* are all estimated to provide a best fit to experimental results, as well as a *V_i_*, the initial virus concentration immediately following inoculation. Table S2 shows the fit values of the model parameters at each experimental temperature. Figure S1A shows the average concentration of virus in the infected guinea pigs as a function of time at 5*°*C and 20*°*C; see Figure 2 in the main text for the comparison at 5*°*C and 30*°*C*.*

## References

1. Baccam P, Beauchemin C, Macken CA, Hayden FG, Perelson AS (2006) Kinetics of influenza A virus infection in humans. J Virol 80: 7590–7599.
2. Beauchemin CA, Handel A (2011) A review of mathematical models of influenza A infections within a host or cell culture: lessons learned and challenges ahead. BMC Public Health 11S1:S7.
3. Murphy BR, Rennels MB, Douglas G, Betts RF, Couch RB, et al. (1980) Evaluation of Influenza-A-Hong-Kong-123-77 (H1n1) Ts-1a2 and cold-adapted recombinant viruses in seronegative adult volunteers. Infect Immun 29:348–355.
4. Parent RA (1992) Treatise on Pulmonary Toxicology, Volume 1: Comparative Biology of the Normal Lung. New York: CRC-Press. 848 p.
