# Supplementary material for: A Comprehensive Breath Plume Model for Disease Transmission via Expiratory Aerosols
Source: PLoS One. 2012 May 15;7(5):e37088. doi: 10.1371/journal.pone.0037088 (PMC3352828; doi:10.1371/journal.pone.0037088)
Supplement: Text S2 — Justification for Neglecting Gravity. (DOC) [file pone.0037088.s007.doc]

**Text S2**

**Justification for Neglecting Gravity**

If the expiratory drops are sufficiently small and the airflow magnitude sufficiently large, then the influence of gravity may be neglected. A quantitative estimate of the influence of gravity is provided by Faxen’s law [1], which relates the actual droplet velocity *dz/dt* to both the gravitational force and the drag force exerted by a background airflow of magnitude *u_z_*,

$6\pi\nu a\frac{dz}{dt}=6\pi\nu au_{z}+V_{0}\Delta\rho g$ ***(S2)***

where *ν* = .018 cP is the dynamic viscosity of air and *Δρ* =.998 g/cm^3^ is the density difference between liquid water and air [2]. Note that higher order corrections to the drag force [1] are omitted here for simplicity. For droplets with size *a* ≈ 8 µm, (which is the largest size of droplets observed by Gustin *et al.*, cf. Figure S1B), substitution of characteristic values into Equation S2 shows that the drag force overwhelms gravity if *u_z_* ≫ 7 cm/s. For 1 µm droplets, the drag force predominates if *u_z_* ≫ .1 cm/s. Since the vertical component of the turbulent velocity is on the order of ≈ 1 cm/s, it is clear that gravity is negligible for droplets smaller than 1 µm, which comprise more than 85% of the droplets. For the larger droplets, the effect of gravity is more pronounced, but since the horizontal component of the airflow velocity is ≈ 10 cm/s, a simple calculation demonstrates that the largest droplets will not sediment to the bottom of the cage until they have already traveled more than ≈ 50 cm downstream, likely past the naïve guinea pig in the adjacent cage for the experiments under consideration here. For animal species where droplets larger than ≈ 8 µm are released (such as primates or humans), inclusion of both the drag and gravitational forces will be necessary.

## References

1. Russel WB, Saville DA, Schowalter WR (1989) Colloidal Dispersions. Cambridge: Cambridge University Press. 544 p.
2. Mills AF (1995) Fundamentals of Physiology: A Human Perspective. New York: CRC-Press. 1240 p.
