# Supplementary material for: A Comprehensive Breath Plume Model for Disease Transmission via Expiratory Aerosols
Source: PLoS One. 2012 May 15;7(5):e37088. doi: 10.1371/journal.pone.0037088 (PMC3352828; doi:10.1371/journal.pone.0037088)
Supplement: Text S3 — Respiratory Particle Size Distribution. (DOC) [file pone.0037088.s008.doc]

**Text S3**

**Respiratory Particle Size Distribution**

Infected individuals expel influenza-laden respiratory particles during regular breathing. The size distribution of these particles was explored in detail by Gustin *et al.* [1], who used an aerodynamic particle sizer (APS) coupled with a two-stage cascade impactor to study respiratory aerosols released by ferrets intranasally inoculated with influenza (Pan99).

Here we use the distribution obtained by Gustin *et al.* for a thirty minute sampling of normal ferret breathing. Measurements were taken days 2, 4, and 6 post-inoculation, and for simplicity the values for each day have been averaged to give an estimated count per particle radius. Figure S1B shows the averaged particle distributions. Droplets are grouped into size bins and represented as discrete points. More than 50% of the particles have a radius less than .5 µm, and greater than 95% have a radius less than 5 µm. The number of deposited pathogens can be calculated for each bin and summed to find the total. The probability for infection is then calculated by inserting the resulting total number of deposited pathogens into the Poisson infection probability (cf. Equation 11 in the main text).

## References

1. Gustin KM, Belser JA, Wadford DA, Pearce MB, Katz JM, et al. (2011) Influenza virus aerosol exposure and analytical system for ferrets. Proc Natl Acad Sci USA 106: 8432-8437.
