# Supplementary material for: A Comprehensive Breath Plume Model for Disease Transmission via Expiratory Aerosols
Source: PLoS One. 2012 May 15;7(5):e37088. doi: 10.1371/journal.pone.0037088 (PMC3352828; doi:10.1371/journal.pone.0037088)
Supplement: Text S4 — Comparison with Steel et al. experiments. (DOC) [file pone.0037088.s009.doc]

**Text S4**

**Comparison with Steel *et al.* Experiments**

The probability model can be further tested using the recently published findings of Steel *et al.* [1] who repeated airborne transmission experiments with guinea pigs in the Caron model 6030 environmental chamber. Their study explored the effect of ambient conditions on aerosol transmission of a strain of H1N1, influenza A/Netherlands/602/2009 (which we refer to as NL09). The timing of the 2009 H1N1 pandemic was not consistent with the usual seasonal occurrence of influenza, so Steel *et al.* looked at the effect of temperature and humidity on airborne transmission of both NL09 and Pan99.

Figure S2 displays the average viral titer measurements from the inoculated animals for the two temperatures studied, 20°C (Figure S2A) and 30°C (Figure S2B), fit to the target cell-limited model [2]. The viral kinetics curves show that both Pan99 and NL09 reach a higher peak viral titer at 20°C, followed by a period of rapid viral decay. At 30°C, the viral kinetics curves have a lower maximum value, and the decay period for Pan99 is slower; there is still measurable virus up to 10 days post-inoculation (p.i.), while at 20°C the viral titers are minimized by the end of the experiment, day 8 p.i. For both temperatures, NL09 viral titers are below detectable levels by day 8 p.i.

Figure S3 shows the contour plots for the transmission experiments performed by Steel *et al*. with their reported infection rates. They found that transmission patterns of Pan99 and NL09 were very similar, and confirmed that both were dependent upon temperature and relative humidity, mirroring the previously published results of Lowen *et al*. [3, 4] Airborne transmission was entirely suppressed at high temperature and high humidity (30°C, 80% RH), and occurred minimally at high temperature and low relative humidity (30°C, 20% RH) and lower temperature and high relative humidity (20°C, 80% RH). Transmission was efficient at lower temperature and moderate relative humidities (20°C, 65% RH) [1]. Figure S3 illustrates that our transmission model is consistent with the findings of Steel *et al.* for the ambient conditions tested (Figure 4A-B, E-H), excluding the high temperature and low humidity case (Figure 4E-F). The model also accurately predicts the similarity between the transmission patterns of Pan99 and NL09. In each case, χ, the ratio of the bronchial and nasal pathogen titers, is assumed to be unity. The viral kinetics within the infected animals for each strain are averaged for each experimental temperature, but in the future could similarly be explored for varied temperature and humidity. To our knowledge, the effect of either environmental parameter on the viral kinetics within the infected animals has not been explored.

## References

1. Steel J, Palese P, Lowen AC (2011) Transmission of a 2009 pandemic influenza virus shows sensitivity to temperature and humidity similar to that of an H3N2 strain. J Virol 85: 1400-1402.
2. Baccam P, Beauchemin C, Macken CA, Hayden FG, Perelson AS (2006) Kinetics of influenza A virus infection in humans. J Virol 80: 7590–7599.
3. Lowen AC, Mubareka S, Steel J, Palese P (2007) Influenza virus transmission is dependent on relative humidity and temperature. PLoS Pathog 3: e151. doi:10.1371/journal.ppat.0030151.
4. Lowen AC, Steel J, Mubareka S, Palese P (2008) High temperature (30°C) blocks aerosol but not contact transmission of influenza virus. J Virol 82: 5650–5652.
